# Transactions of the College of Physicians of Philadelphia

**Published:** 1895-02

**Authors:** William J. Taylor

**Affiliations:** Professor of Orthopedic Surgery in the Philadelphia Polyclinic; Attending Surgeon to St. Agnes Hospital; Assistant Surgeon to the Orthopedic Hospital and Infirmary for Nervous Diseases


### TRANSACTIONS OF THE COLLEGE OF PHYSICIANS OF PHILADELPHIA.

### SECTION OF GENERAL SURGERY.

STATED MEETING, November 9, 1894.
DR. JOHN B. ROBERTS, Chairman, in the Chair.

### WOOD-PULP SPLINTS AND SPLINT-MAKING MATERIAL AS SUGGESTED BY DR. EDWARD A. TRACY, OF BOSTON.

BY WILLIAM J. TAYLOR, M. D.,

Professor of Orthopedic Surgery in the Philadelphia Polyclinic; Attending Surgeon to St. Agnes Hospital; Assistant Surgeon to the Orthopedic Hospital and Infirmary for Nervous Diseases.

I have here a number of splints made from a new material, the invention of Dr. Edward A. Tracy, of Boston, and which he was kind enough to send me, along with some of the sheets of the material itself. You have probably all seen the article by Dr. Tracy in *The Medical News*, of March 17, 1894, entitled "A Brief Splint-Technology for Surgeons." When I read the article I was so much impressed with the probable value of the material for splint-making that I wrote to him, and it is in answer to this letter that he sends me these samples.

All of these splints were made by him and have been in actual use at the Boston City Hospital. He writes me "that the results to be gotten will repay the trouble of acquiring the proper technique—and this trouble is but slight."

The material is best described by Dr. Tracy's article, just referred to, and of which the following is an abstract: "The basis of the material is wood-pulp, made preferably from the crushed fibre of the poplar tree, and rolled in such fashion that the broken fibres intertwine in every direction and loosely, so that an increase of plasticity is thus given the product. These sheets are further strengthened by having a fabric introduced between the layers of the pulp, or by interweaving with the short, crushed wood-fibre, a long jute, or other tough fibre.

"The sheets are rolled of different thicknesses, and are numbered one, two and three, according to their thickness in millimeters.

"The chief characteristics of this material are stiffness or rigidity when dry, and plasticity when moist. Its rigidity can be increased *ad libitum* by the use of a silicate solution as a moistener. Its plasticity has a limit, but this is rarely reached except when moulding the material over certain complex curved surfaces. Besides the foregoing characteristics, the material possesses that desideratum of a splint-material—extreme lightness. Its cheapness also deserves a passing mention.

"Water or a stiffening solution can be used to moisten the material. If water is used the splint must be protected from the perspiration, lest it soften, by a covering of oiled-silk, or paper, or best by shellac. A stiffening solution with several qualities to recommend it is that of silicate of potassium; silicate of soda is almost as serviceable. Any desired degree of rigidity can be imparted to a splint by using this solution—the amount of rigidity depending upon the strength of the solution. Dextrine in the proportion of 8 ounces to the pint of water adds some tenacity besides stiffness. A splint made with its aid can be moistened with water and re-moulded—quite an advantage in many cases.

"In using this material the aim should be to get barely sufficient moisture to render the material semi-plastic. If more moisture is absorbed it becomes difficult to maintain the moulded splint in the desired shape while drying, and it also unnecessarily lengthens the time required to dry it. The best manner of moistening the splint-blank is to apply the fluid used on each side of it alternately by means of a flat paste-brush. A little practice will enable one to judge the precise amount of moistening best suited to the purpose. The time required for drying the moulded splints varies from ten to forty minutes, according to the thickness of the material used; any source of heat can be employed, a kitchen fire being serviceable, and nearly always at hand.

"While drying the splint after it is moulded to fit the part, it is well to wind a bit of yarn or string around it to aid in retaining its shape until it is sufficiently hard to keep its form."

He then describes in detail the application of splints made from this material to different portions of the body, including the head, the trunk, and the upper and lower limbs.

As I have only just recently received the material, my personal knowledge of the technique is limited, but I can readily see the ease with which the sheets can be moulded to fit inequalities in the limbs. The extreme lightness of the splint will be of the greatest service in many cases, and will be much more comfortable to the patient than the heavier and more cumbersome splints in common use. Then, too, by coating the splint with a silicate solution, it becomes impervious to moisture and can be washed daily with an antiseptic solution.

